# Genetic variation of the Chilean endemic long-haired mouse *Abrothrix longipilis* (Rodentia, Supramyomorpha, Cricetidae) in a geographical and environmental context

**DOI:** 10.7717/peerj.9517

**Published:** 2020-07-16

**Authors:** Lourdes Valdez, Marcial Quiroga-Carmona, Guillermo D’Elía

**Affiliations:** Instituto de Ciencias Ambientales y Evolutivas, Universidad Austral de Chile, Valdivia, Chile

**Keywords:** Abrotrichini, Chile, Ecological niche modelling, Historical demography, Phylogeography, SNPs, Population genomics

## Abstract

Quaternary climate and associated vegetational changes affected the fauna of the Chilean Mediterranean ecosystem. Here we studied the genetic variation of the long-haired mouse, *Abrothrix longipilis*, a sigmodontine rodent endemic to this area. Within an environmentally explicit context, we examined the geographic distribution of the genetic diversity and demographic history of the species based on sequences of the mitochondrial Cytochrome-b gene of 50 individuals from 13 localities and a large panel of single nucleotide polymorphisms of 17 individuals from 6 localities. The gene genealogy of *A. longipilis* revealed three intraspecific lineages that are allopatric and latitudinally segregated (northern, central, and southern lineages) with an estimated crown age for the whole species clade of 552.3 kyr B.P. A principal component analysis based on 336,596 SNP loci is in line with the information given by the the mitochondrial gene genealogy*.* Along its complete distributional range, *A. longipilis* showed patterns of isolation by distance and also isolation by environment. The general pattern of historical demography showed stability for most intraspecific lineages of *A. longipilis.* Northern and central lineages showed signals of historical demographic stability, while the southern lineage showed contrasting signals. In agreement with this, the niche models performed showed that in the northern range of *A. longipilis*, areas of high suitability for this species increased towards the present time; areas of central range would have remained relatively stable, while southern areas would have experienced more change through time. In summary, our study shows three distinct allopatric lineages of *A. longipilis*, each showing slightly different demographic history.

## Introduction

Central Chile (30°–36°S) harbors one of the five Mediterranean-type ecosystems of the World (Cowling et al., 1996). The Chilean Mediterranean zone represents a transition between the Atacama Desert, one of the World’s driest deserts, to the north and the mixed-deciduous and temperate Valdivian forest southwards. The macroclimate in the region has hot dry summers and cold rainy winters, with annual precipitation varying, north to south, from less than 200 mm to 700 mm (Armesto & Martínez, 1978). Two parallel mountain ranges, the Coastal Pacific cordillera and the Andes, are oriented north to south and separated by an 80–100 km wide Central depression. Main vegetation types in the northern Mediterranean area include dry xerophytic thorn scrublands and evergreen sclerophyllous communities, while southern areas are dominated by deciduous forest (Armesto, Arroyo & Hinojosa, 2007; Villagran, 1995).

The climate and vegetation of central Chile experienced changes during the Quaternary glacial cycles. As the climate turned colder and wetter and the glaciers advanced along the Andes, the Mediterranean-type formations got restricted towards its current northern limits. During the last glacier advance, ice coverage in the Central Andes reached elevations as low as 1.200 m above sea level, outwashing 12,000 years ago (Heusser, 1990). During the Pleistocene-Holocene transition, warmer-drier conditions favored the expansion of Mediterranean formations toward the south and also the recolonization of higher altitudinal areas (Paskoff, 1977; Villagrán & Varela, 1990; Markgraf, 1993).

It is well known that climate and associated vegetational changes affected the local fauna. For instance, a large comparative study conducted with the assemblage of Patagonian small rodents showed that responses to Quaternary climate varies among species (Lessa, D’Elía & Pardinas, 2010; see also Cañón et al., 2010; Alarcón et al., 2011). No such comparative study has been conducted for the mammal fauna of Central Chile; notwithstanding, it has been proposed that Pleistocene refugial areas for several small mammals, including sigmodontine rodents, occurred in Central Chile. This scenario was advanced for species that are more or less restricted to Mediterranean environments, such as *Octodon degus* (Valladares, 2009), *Phyllotis darwini* (Gutiérrez-Tapia & Palma, 2016), and *Spalacopus cyanus* (Opazo et al., 2008), as well as for the widely distributed species *Oligoryzomys longicaudatus* (Palma et al., 2012) and *Abrothrix olivacea* (Smith, Kelt & Patton, 2001). In terms of the approximate location of refugial areas and the direction of postglacial colonization in the area, as in Patagonia, each species shows its own pattern. For instance, for *O. longicaudatus* a coastal refugium (approx. at 39–40°S), from which this species would have colonized towards the north and south, was proposed (Palma et al., 2012). Also from a coastal refugium at 29°30′–30°S, *Octodon degus* would have a southward colonization (Valladares, 2009). A different pattern of postglacial colonization, from the Andes towards lowland and coastal areas, was inferred for *Spalacopus cyanus* (Opazo et al., 2008). *Phyllotis darwini* would have colonized from a 31–35°S located refugium northwards and towards the Andes (Gutiérrez-Tapia & Palma, 2016); meanwhile, colonization southwards and towards the Andes from ca. 40°S was proposed for *Abrothrix olivacea* (Smith, Kelt & Patton, 2001). Recently, Palma et al. (2017) suggested for *Phyllotis darwini* and *Abrothrix olivacea* postglacial expansion from the lowlands and coastal areas towards the Andes; however, as this study considers a very small fraction of the whole species distributions (i.e., a sampled area restricted to 32° and 33° for species ranging from ca. 23° to 39° and ca. 18° to 55°, respectively) and as it has not been shown that the genetic variants of the studied area form a monophyletic group, the value of such inference is dubious.

Given that few species in the region have been studied in their entirety, it is important to examine additional species because there is a species-specific response to Quaternary climate among the small mammals from Central Chile. Here we focus on understanding the genetic footprints of these changes on populations of the long-haired mouse *Abrothrix longipilis* (Waterhouse, 1837). This sigmodontine mouse, described based on a specimen collected by Charles Darwin in the Chilean coast of Coquimbo, is endemic to Central Chile. The known geographic distribution of *A. longipilis* ranges from Coquimbo (ca. 30°S) to the coastal and Andean areas of the Maule region (ca. 35°S) in the south; the southern limits of the distribution is unclear, as there is a large geographic area (ca. 33.5°S and 36.5°S) where it is unclear if *A. longipilis* or *A. hirta* occur (see Teta & Pardiñas, 2014). No study has assessed the degree and pattern of the genetic variation of the Mediterranean *A. longipilis*.

In this study, with an environmentally explicit approach, we examine the geographic distribution of the genetic diversity of *Abrothrix longipilis*. We evaluated the pattern of genetic variation and the demographic history of the species based on sequences of the mitochondrial Cytochrome-b (*Cytb*) gene and a large panel of single nucleotide polymorphisms (SNPs). We also tested for patterns of isolation by distance and isolation by environment. We discuss our findings in the context of available antecedents to improve our understanding of the historical dynamics of the small mammal fauna in Central Chile.

**Figure 1:**
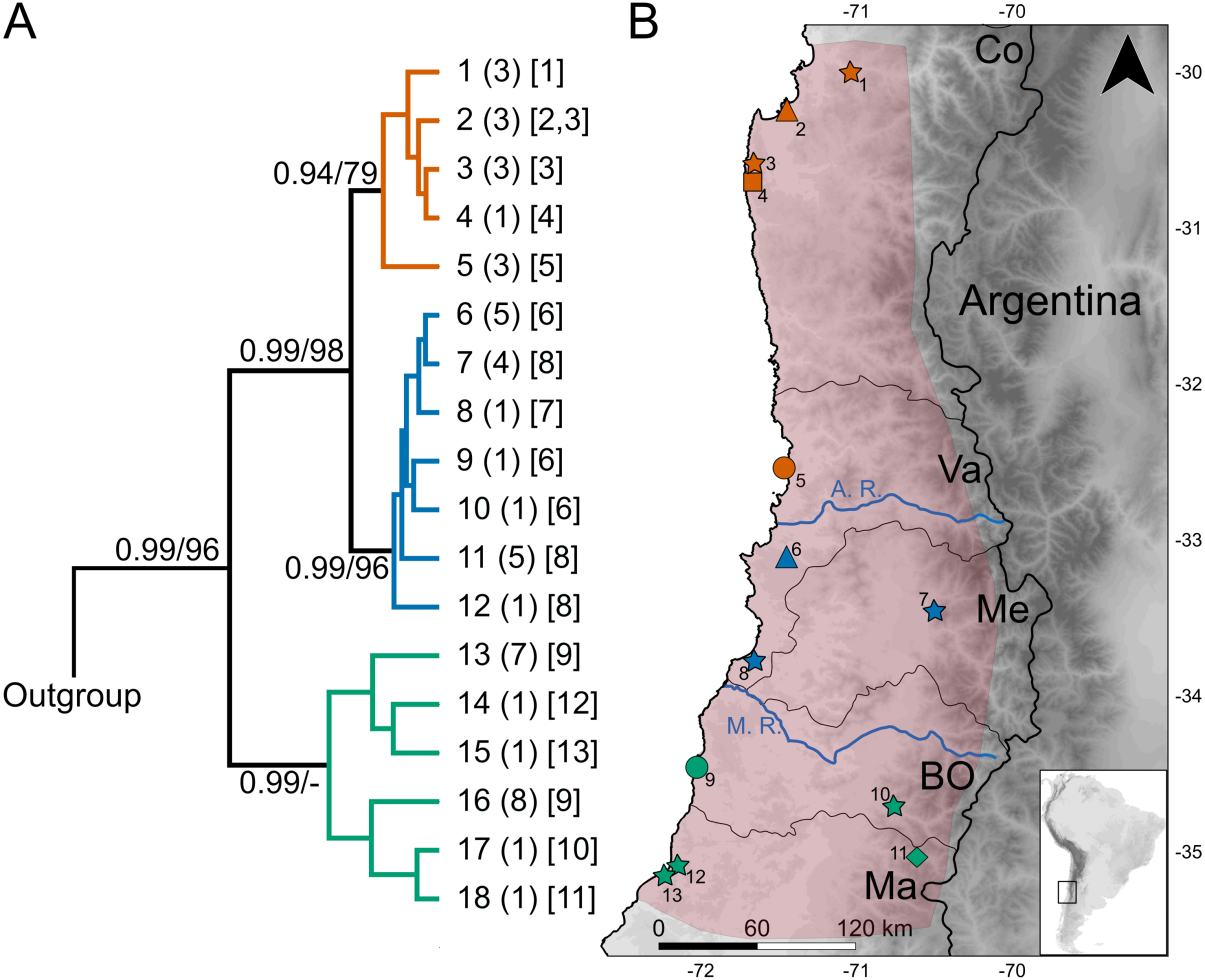
Geographic variation of the mitochondrial diversity of *Abrothrix longipilis*. (A) The tree shows the genealogical relationship, reconstructed via Bayesian inference, of 18 *Cytb* haplotypes of *Abrothrix longipilis.* Support values correspond to posterior probability and the bootstrap proportions found in a Maximum Likelihood analysis. The three numbers in tip labels are: first, haplotype number (as in Table 1), second, the number of specimens sharing each haplotype, and third, the locality number/s where each haplotype was found. (B) Map of north-central Chile depicting in pink the known distribution of *Abrothrix longipilis* as expanded in this study (localities 9-13). Chilean Regions are indicated as Co = Coquimbo, Va = Valparaíso, Me = Metropolitana, BO = Bernardo O’Higgins, and Ma= Maule. The courses of the rivers Aconcagua (A. R.) and Maipo (M. R.) are shown in blue. Collecting localities of the specimens analyzed here are numbered as in Table 1. Colors and symbols are the same used in Fig. 3; these localities; those localities without specimens used in the genomic analysis are depicted with stars.

## Materials & Methods

### Specimen sampling and data collection

Genetic analyses were based on two datasets. One of these datasets consists of DNA sequences of the first 801 bp of the mitochondrial *Cytb* gene gathered from 50 individuals of *Abrothrix longipilis*; these specimens were collected at 13 localities along most of distributional range of the species (Fig. 1; Table 1). Seventeen of these *Cytb* sequences were downloaded from GeneBank. The other 33 were generated from specimens collected by us under permits granted by the Chilean Servicio Agrícola y Ganadero numbers 1896/2017, 4532/2017, 1896/2017, 1231/2017, 807/2018 and 1816/2019. These specimens are housed at the Colección de Mamíferos de la Universidad Austral de Chile. Animal handling was approved by the Comité de Bioética “Uso de animales en la Investigación”, Universidad Austral de Chile (UACh). The second dataset contains 336,596 SNP loci from 17 individuals of *A. longipilis* collected at 6 localities (numbers 2, 4, 5, 6, 9 and 11 in Fig. 1 and Table 1). These specimens are among those collected by us and all were sequenced for the *Cytb* gene.

**Table 1:** Sampling of specimens of *Abrothrix longipilis* included in this study. Localities are numbered as in Fig. 1. For each specimen included it is provided: specimen voucher, Genbank accession number, the number assigned to the recovered haplotype, and the source of the sequence. Specimens from which SNP data was generated are identified with an asterisk.

#	Locality	Specimen, GenBank accession, haplotype	Reference
1	Región de Coquimbo, Elqui, La Serena, Fundo el Hinojal, −30.000189, −71.039969	NK106120, GU564005, 1; NK106122, GU564006, 1; NK106134, GU564007, 1	Palma et al. (2014)
2	Región de Coquimbo, Coquimbo, Tongoy, Estero Tongoy, −30.280217, −71.460617	UACH8118*, MN275210, 2; UACH8119*, MN275209, 2; UACH8120*, MN275208, 2	This paper
3	Región de Coquimbo, Limarí, Ovalle, Parque Nacional Fray Jorge, −30.638408, −71.654608	NK105517, GU564008, 3; NK105520, GU564009, 2; NK105528, U564010, 3	Palma et al. (2014)
4	Región de Coquimbo, Limarí, Ovalle, LimaríSur, −30.73381, −71.68641	UACH8129*, MN275195, 4	This paper
5	Región de Valparaíso, Petorca, Zapallar, −32.540333, −71.464383	UACH8140*, MN275226, 5; UACH8141*, MN275224, 5; UACH8142, MN275225 *, 5	This paper
6	Región de Valparaíso, Valparaíso, La Cantera, Reserva Nacional Lago Penuelas, −33.16988, −71.45735	UACH8121*, MN275227, 6; UACH8122, MN275201, 6; UACH8123, MN275203, 9; UACH8124, MN275202, 6; UACH8125, MN275200, 6; UACH8126, , MN275198 10; UACH8127, MN275197, 6	This paper
7	Región Metropolitana, San Carlos de Apoquindo, −33.46896389, −70.48848056	NK105006, GU564011, 8	Palma et al. (2014)
8	Región de Valparaíso, San Antonio, Santo Domingo, Fundo la Ventolera, −33.745875, −71.652328	NK105989, GU564012, 11; NK105993, GU564013, 7; NK106002, GU564014, 7; NK106006, GU564015, 11; NK106007, GU564016, 12; NK106009, GU564017, 7; NK106013, GU564018, 11; NK106015, GU564019, , 11; NK106016, GU564020, 11; NK106017, GU564021, 7	Palma et al. (2014)
9	Región de O’Higgins, Cardenal Caro, Cáhuil, −34.4604, −72.02465	UACH8093, MN275223, 13; UACH8095, MN275222, 13; UACH8097, MN275199, 13; UACH8101, MN275221, 13; UACH8105*, MN275218, 13; UACH8115, MN275206, 13; UACH8116, MN275207, 13; UACH8107*, MN275215, 16; UACH8108*, MN275214, 16; UACH8109, MN275216, 16; UACH8110, MN275217, 16; UACH8111, MN275212, 16; UACH8112, MN275213, 16; UACH8113*, MN275204, 16; UACH8114*, MN275205, 16 , UACH8102*, UACH8104*, GD1918*	This paper
10	Región de O’Higgins, Cochalhua, San Fernando - Por camino, 3.8 Km de la Rufina, −34.735267, −70.727083	UACH8088, MN275211, 17	This paper
11	Región del Maule, Curicó, Romeral, Río Teno, −35.04697, −70.5984	UACH8128*, MN275196, 18	This paper
12	Región del Maule, Talca, Curepto, Ruta J52K Km 4.21 −35.080167, −72.167217	UACH8117, MN275219, 14	This paper
13	Región del Maule, Talca, Constitución, Humedales de Putú, Ruta k24, km 28.4 −35.162933, −72.253533	UACH8087, MN275220, 15	This paper

### Sequence acquisition, alignment, and analysis

Seventeen *Cytb* sequences were downloaded from GenBank (Table 1), while 33 were newly generated following the protocol outlined by D’Elía & Pardiñas (2004). Amplicons were sequenced at an external DNA sequencing service (Macrogen, Seoul, South Korea). Reads were edited using CodonCode (Codon-Code, Dedham, Massachusetts). The character primary homology was established aligning the *Cytb* sequences with Clustal W (Thompson, Higgins & Gibson, 1994) that is in Mega 7 (Kumar, Stecher & Tamura, 2016). The alignment was visually inspected in search of internal codon stops and reading frame shifts; no corrections were needed. The software DNAsp (Rozas et al., 2017) was used to calculate haplotype and nucleotide diversity indexes. One representative of each haplotype class was used in genealogical analyses, i.e., a non-redundant matrix using DNAsp. Using this matrix, a neighbor-joining tree was constructed with Mega 7 to visually corroborate the absence of segregating sites within haplotype clases. Sequences of *Cytb* from the congeneric species *Abrothrix hirta* (GenBank accession GU564080), *A.manni* (KJ614629, KP665998), *A. sanborni* (KP666004), *A. lanosa* (KP666018), *A. illutea* (KJ614622), *A. olivacea* (AF027306, HM167800), and *A. andina* (AF108671), and the abrotrichines *Paynomys macronyx* (U03533) and *Geoxus valdivianus* (U03531) were used to conform the outgroup. The best-fit model of nucleotide substitution was detected based on Bayesian Information Criterion using jModeltest2 (Darriba et al., 2012). The selected model, TMP2, was set in genealogical reconstruction using two approaches, Maximum Likelihood (ML) and Bayesian inference (BI). The ML tree was inferred using IQ-TREE (Nguyen et al., 2015) at the IQ-TREE web server (Trifinopoulos et al., 2016) with 100 unsuccessful iterations, and 1,000 replicates of ultrafast bottstrap for branch support estimation (BS; Minh, Nguyen & Von Haeseler, 2013. The BI analysis was conducted using BEAST2 (Bouckaert et al., 2014). Tree topology, substitution model parameters, and dates for each cladogenetic event were estimated simultaneously. A birth-death process (Stadler, 2009) with an initial random tree, and other parameters set as default were used. The TMP2 substitution model was implemented with AC=AT, AG=CT, CG=GT and equal base frequency with empirical base frequencies and four gamma categories. A strict-model clock with a substitution rate of 0.003 was used (Lessa, D’Elía & Pardiñas, 2010). Two independent runs, consisting of 100 × 10^6^ MCMC iterations, sampling change every 2000 generations, were conducted. The first 20% of the samples were discarded as burnin. Convergence to stable values was checked using Tracer v. 1.7.1 (Rambaut et al., 2018), obtaining an effective sample size (ESS) greater than 200 for all parameters. Tree and log files were combined using LogCombiner included in BEAST2. A maximum clade credibility (MCC) was calculated using TreeAnnotator in BEAST2 to display mean node ages and highest posterior density (HPD) intervals (95% upper and lower) for each node. Genetic differentiation was estimated based on p-distances, which was calculated using Mega 7.

Historical demography was assessed with Arlequin 3.5 (Excoffier & Lischer, 2010). The total haplotype sample of *Abrothrix longipilis*, as well as haplotype samples grouped according to clades obtained in the genealogical analyses (see below), were subjected to analyses of demographic history, including mismatch distribution (Rogers & Harpending, 1992), Tajima’s (1989) and Fu’s (1996) tests of departure from neutrality, and coalescent-base Bayesian skyline plots (Drummond et al., 2005). A population in demographic equilibrium draws a multimodal mismatch distribution, meanwhile, a population in recent demographic expansion yields a unimodal curve (Rogers & Harpending, 1992). The raggedness index (hr) was used to quantify the smoothness of the mismatch distribution; a significant *p*-value is indicative of recent population expansion (Harpending, 1994). Negative and significant values of Tajima’s D and Fu’s F indicate population expansion. Changes in the effective population size through time was estimated using BEAST2 and displayed in Bayesian skyline plots (Drummond et al., 2005). Frequencies and nucleotide substitution were estimated from the dataset; the best fitting model was selected based on Bayesian Information Criterion. The package Model Selection v.1.01 was used to select the clock model; 100 million generations of MCMC was let run, sampling every 1,000 iterations. The output was analyzed with Tracer. The substitution rate of 0.03 was used for *Cytb* gene. This estimation was made for *A. longipilis*, the sister species of *A. hirta* (Lessa, D’Elía & Pardiñas, 2010).

### Transcriptome-derived SNP calling and PCA

SNP data was obtained based on RNA sequencing. Total RNA from 17 individuals, which were also included in the *Cytb* based analyses, were extracted from kidney tissue using the RNeasey mini kit from Qiagen; mRNA-enrichment was performed using Ilumina TruSeq RNA sample preparation kit. Paired-end sequencing (2 × 10^1^ bp per sample) of cDNA from these libraries were performed under Illumina Hiseq 2000 platform. Raw data were trimmed for low quality bases using TrimGalore (http://www.bioinformatics.babraham.ac.uk/projects/trim_galore/). Sequences of Ribosomal RNA were removed from the dataset after read mapping against sequences of rRNA from Rodentia available at GenBank (https://www.ncbi.nlm.nih.gov/nuccore/) using Bowtie2.2.6 (Langmead & Salzberg, 2012). After these filters, remaining reads were assembled de novo using Trinity version 2.8.5 (Grabherr et al., 2011).

SNP calling was conducted using GATK (McKenna et al., 2010), following the protocol recommended by these authors for RNAseq data (see the user Guide at https://software.broadinstitute.org/gatk). Duplicated reads were eliminated from the database using Picard (Wouters et al., 2002). Individual vcf files containing SNP variants were merged into a single file using BCFtools (Danecek & McCarthy, 2017). SNP loci were filtered to include only those sites present in all 17 individuals; linked loci were discarded by including only the first SNP called in a given read. Hardy-Weinberg equilibrium was estimated for each locus using VCFtools (Danecek & McCarthy, 2017); given the large amount of acquired SNPs we took a conservative strategy and excluded from the dataset those sites that deviated from equilibrium below the threshold of 0.05. In order to explore the genetic structure, a Principal component analysis was conducted using PLINK v.1.9 (Zheng et al., 2012).

### Genetic variation across the geographic and climatic space

Patterns of isolation by distance (IBD) and isolation by environment (IBE) were tested using Mantel tests for correlation of matrices as implemented in the R package vegan (Oksanen et al., 2019). IBD was assessed by testing for correlation of matrices of geographic and genetic distances among localities (Tables S1 and S2). Geographic distances among recording localities were measured in kilometers using the R package sp (Pebesma & Bivand, 2005; Pebesma et al., 2013). Genetic distance between local sample pairs was measured in terms of p-distance in sequences of the gene *Cytb* using Mega 7. IBE was tested using the genetic distance matrix described above and a matrix of climatic dissimilarity based on Gower distance (see Gower, 1971; Legendre & Legendre, 1998) estimated among sampling localities. This climatic dissimilarity matrix (Table S3) was calculated based on values registered in each locality for 19 bioclimatic variables obtained from the WorldClim 1.4. database (Hijmans et al., 2005). Climatic data of each locality was extracted using the “extract” function of the raster package (Hijmans et al., 2015). This climatic data was also used to compare climatic attributes of the geographic areas occupied by the main lineages (see below) of *A. longipilis*. With this purpose, a Principal Component Analysis (PCA) was performed using the package FactoMineR (Husson et al., 2020). For this analysis, only localities from where specimens of *A. longipilis* were sequenced were considered (Table 1 and Table S5). Previously, localities were grouped a priori according to clade assignation in the *Cytb* gene genealogy (see below). Values of each bioclimatic variable (Table S5) were employed to construct a correlation matrix since these variables are expressed in different measurement scales (Borcard, Gillet & Legendre, 2018). Additionally, similarity among localities was evaluated employing a cluster analysis based on Gower distance estimates between pairs of localities. All analyses were conducted using R version 3.5.1 (http://www.r-project.org/index.html).

### Ecological niche modelling

Ecological Niche Models (ENM) were built to infer historical changes in size and location of the environmental suitable areas available for *Abrothrix longipilis* in central Chile. The maximum entropy method was performed with MaxEnt v. 3.3.3k (Phillips, Anderson & Schapire, 2006) using data from 13 sampling localities recorded in this study and 17 localities obtained from Vertnet database (http://www.vertnet.org). To reduce the possible effects of spatial autocorrelation, these 30 records were spatially filtered with the R package spThin (Aiello-Lammens et al., 2015), obtaining a final dataset of 27 localities of presence (Table S6) separated by more than 10 km of distance.

Nineteen bioclimatic variables obtained from WorldClim 1.4., with a spatial resolution of 30 arc-second, were used as environmental predictor variables in ENM. Each model was trained and calibrated in a geographic region delimited based on the observed geographic distribution of *Abrothrix longipilis*, following Anderson & Raza (2010) and Barve et al. (2011). This region was delimited adjusting a minimum convex polygon to the filtered presence records of *A. longipilis* and adding a geographic buffer of 50 km (∼0.5°). This procedure was carried out with the R package “dismo” (Hijmans et al., 2017). Estimation of optimal model performance and complexity was completed with the package ENMeval (Muscarella et al., 2014), with values of regularization multiplier set to vary from 0.5 to 4.0 and increments of 0.5, and combining feature classes Linear (L) and Linear-Quadratic (LQ) given the number of occurrences of *A. longipilis* (Phillips & Dudík, 2008).

The method employed in the ENM evaluation was “block”, as recommended for ENM implementations that require transference between temporal or geographic scenarios (Radosavljevic & Anderson, 2014). Occurrence data was randomly partitioned in four sets 100 times. The combinations of values of regularization multiplier and features classes employed resulted in 16 model settings for each run. The final model (i.e., those built with the final setting of MaxEnt selected) was constructed with the combination of regularization multiplier and feature classes that yielded the lowest value of corrected Akaike Information Criteria (AICc). The performance of the model selected as optimal was assessed inspecting omission rate (OR) and the test of Area Under the Curve (AUC). Continuous values of environmental suitability were converted to a categorical representation, to classify each pixel of projection into intervals ranging from 0 to 1, with increments of 0.25 (four categories of environmental suitability in total). This transformation was employed to construct maps of geographic distribution of the abiotically suitable areas for *A. longipilis*, which can be interpreted as a spatial representation of its potential geographic distribution (Soberón & Peterson, 2005) in each climatic scenario evaluated. Final models were projected on the study region corresponding to northern-central Chile (28°70′S, 74.53′W and 36.65°S, 67.96°W) and for climatic scenarios existing during the Last Interglacial (LIG: ∼120–140 kyr B.P.), the Last Glacial Maximum (LGM: ∼22 kyr B.P.), the Mid Holocene (Mid-Holo: ∼6 kyr B.P.), and the current period (1960–1990). Palaeoclimatic data used for projections of ENM in past scenarios were obtained from several general circulation models; for the LIG climate the simulation of arctic climate warmth and ice retreat (Otto-Bliesner et al., 2006) was used, while for LGM and Mid-Holo the models used were the Community Climate System Model (CCSM4; Gent & Danabasoglu, 2011) and the Model for Interdisciplinary Research on Climate (MIROC-ESM; (Watanabe et al., 2010)). These procedures were performed using DIVA-GIS, version 7.5 (Hijmans et al., 2001).

## Results

### Molecular diversity

The dataset of 50 *Cytb* sequences of *Abrothrix longipilis* exhibited 48 variable sites that define 18 haplotypes (Table 1), of which nine were found in more than one specimen. Only one haplotype (#11) was collected at two sampling sites (2 and 3; Fig. 1, Table 1). On average, divergence among localities is 1.6% (range: 0.1%–3.1%). Haplotype and nucleotide diversity for the whole dataset are Hd: 0.928 and Pi: 0.01639, respectively.

### Gene genealogy and divergence times

The topologies recovered by both methods used for gene genealogy reconstruction were similar. The single noteworthy difference is noted below. The Bayesian tree is shown in Fig. 1. The monophyly of *Abrothrix longipilis* was strongly supported (PP = 0.99; BS = 96). Haplotypes of *A. longipilis* formed three main allopatric clades. A northern clade (PP = 0.94; BS = 79) contained haplotypes from localities 1 through 5 (orange clade in Fig. 1) laying in the Coquimbo Region and northern Valparaiso Region; a central clade (PP = 0.99; BS = 96) included haplotypes from specimens collected at localities 6 to 8 (blue clade in Fig. 1) in southern Valparaiso Region and Metropolitana Region, and a southern clade (PP = 0.99) contained haplotypes collected at sites 9 to 13 (green clade in Fig. 1) in O’Higgins and Maule Regions. The main difference between the Bayesian and ML topologies was that in the latter the southern clade was not recovered; its constituent haplotypes fell in a polytomy at the base of the clade of *A. longipilis*. In both ML and Bayesian topologies, the northern and central clades were sister to each other in a strongly supported relationship (PP = 0.99; BS = 98). Mean genetic distance between northern and central clades was 1%, between northern and southern clades is 2.6%, and between central and southern clades was 2.7%.

**Figure 2:**
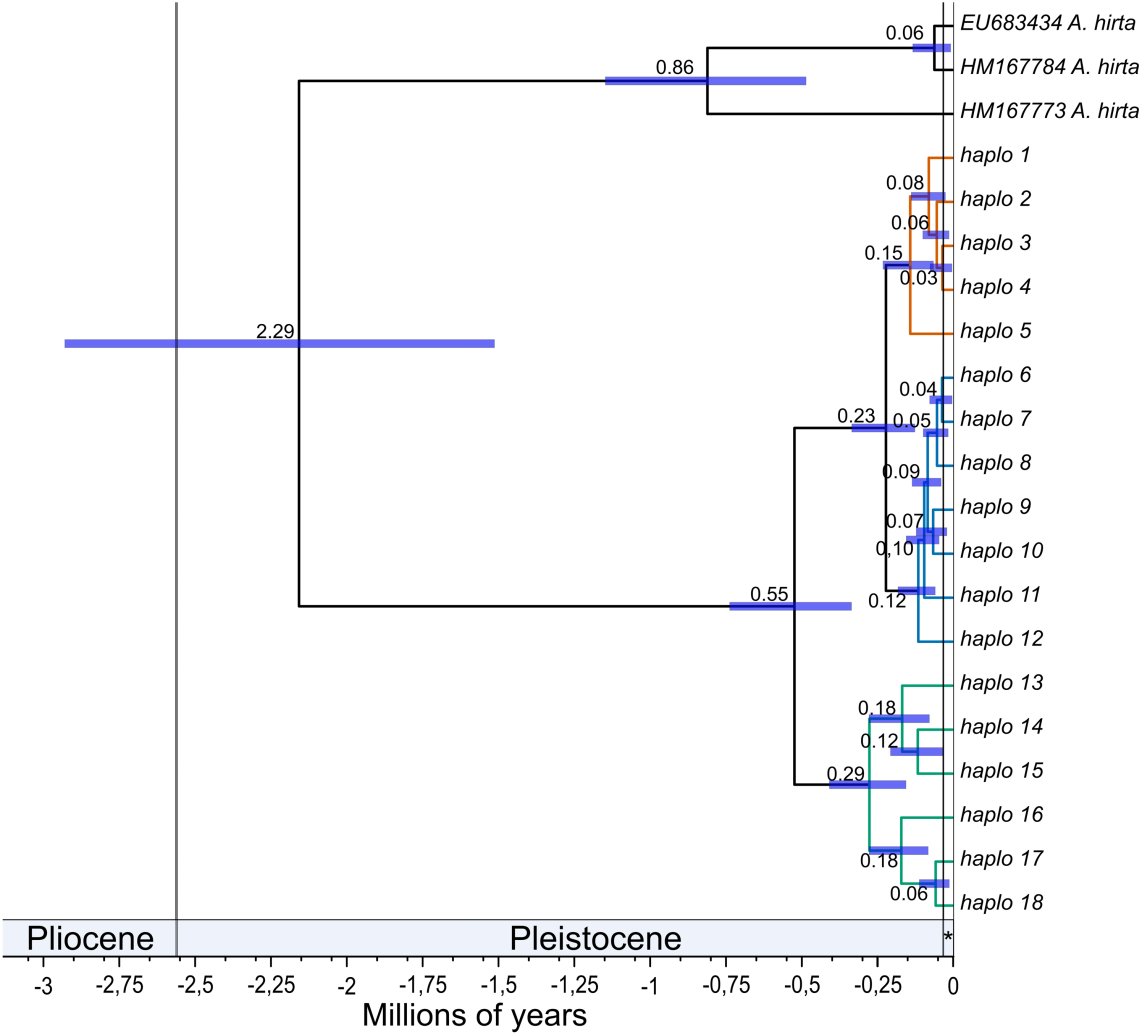
Divergence times among mitochondrial lineages of *Abrothrix longipilis.* Bayesian chronogram showing estimated mean divergence times for distinct intraspecific clades of *Abrothrix longipilis.* Node bars show the 95% confidence interval. Clades are colored as in Fig. 1. Haplotype information is given in Table 1. The Holocene is indicated with an asterisk.

The mean estimated crown age for *Abrothrix longipilis* was 552.3 kyr (with a 95% range of 352.9–772.9). The lineages leading to the northern and central clades diverged from each other at 230 kyr ago (131.4–351.9). Crown ages for the northern, central, and southern clades were 150 (70–240), 110 (60.6 - 190) and 290 kyr (162.5–430.3), respectively. Figure 2 shows these estimated divergence times and those for internal clades of the northern, central and southern clades (see Table S14 for values of each estimate).

### Genetic structure based on SNP variation

**Figure 3:**
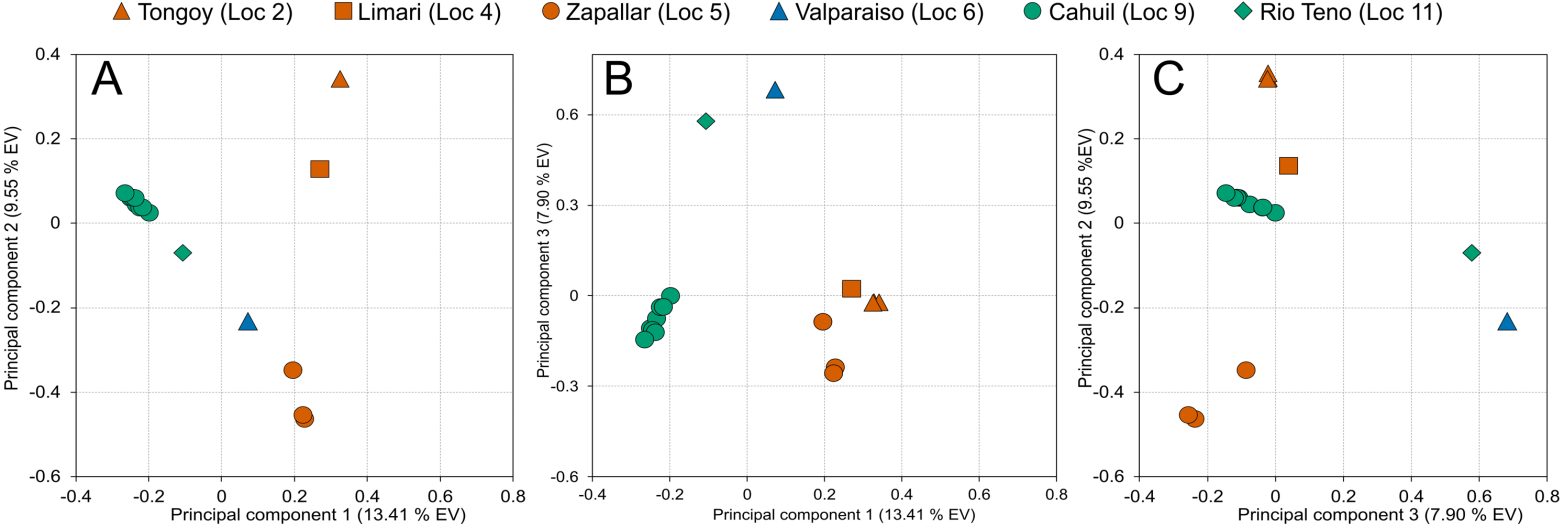
Genetic structure of *Abrothrix longipilis* as revealed by SNP variation. Principal Component Analysis based on 336,596 SNP loci from 17 individuals of *Abrothrix longipilis.* Colors correspond to those of the main mitochondrial lineages and shown in Fig. 1. Locality symbols and numbers correspond to those of Fig. 1 and Table 1.

A total of 575,981 raw SNP loci were obtained based on the transcriptome of 17 individuals of *Abrothrix longipilis.* After filtering (see Materials and methods), 336,596 loci were included in the PCA analysis (Fig. 3, Tables S7–S8). 30.8% of the total observed variation was explained by the first 3 principal components (Table S9). Individuals segregated in the multidimensional space in accordance with the mitochondrial clades into three non-overlapping groups (Fig. 3A). PC1 completely discriminated between northern, central, and southern groups (Figs. 3A, 3B). PC2 and PC3 segregated samples from different collecting sites within clades; i.e., samples from Tongoy, Limarí, and Zapallar within the northern clade (Localities 2, 3 and 5; Figs. 3A, 3B) and Cáhuil and Río Teno within the southern clade (Localities 9 and 11; Fig. 3A–3C).

### Historical demography

**Figure 4:**
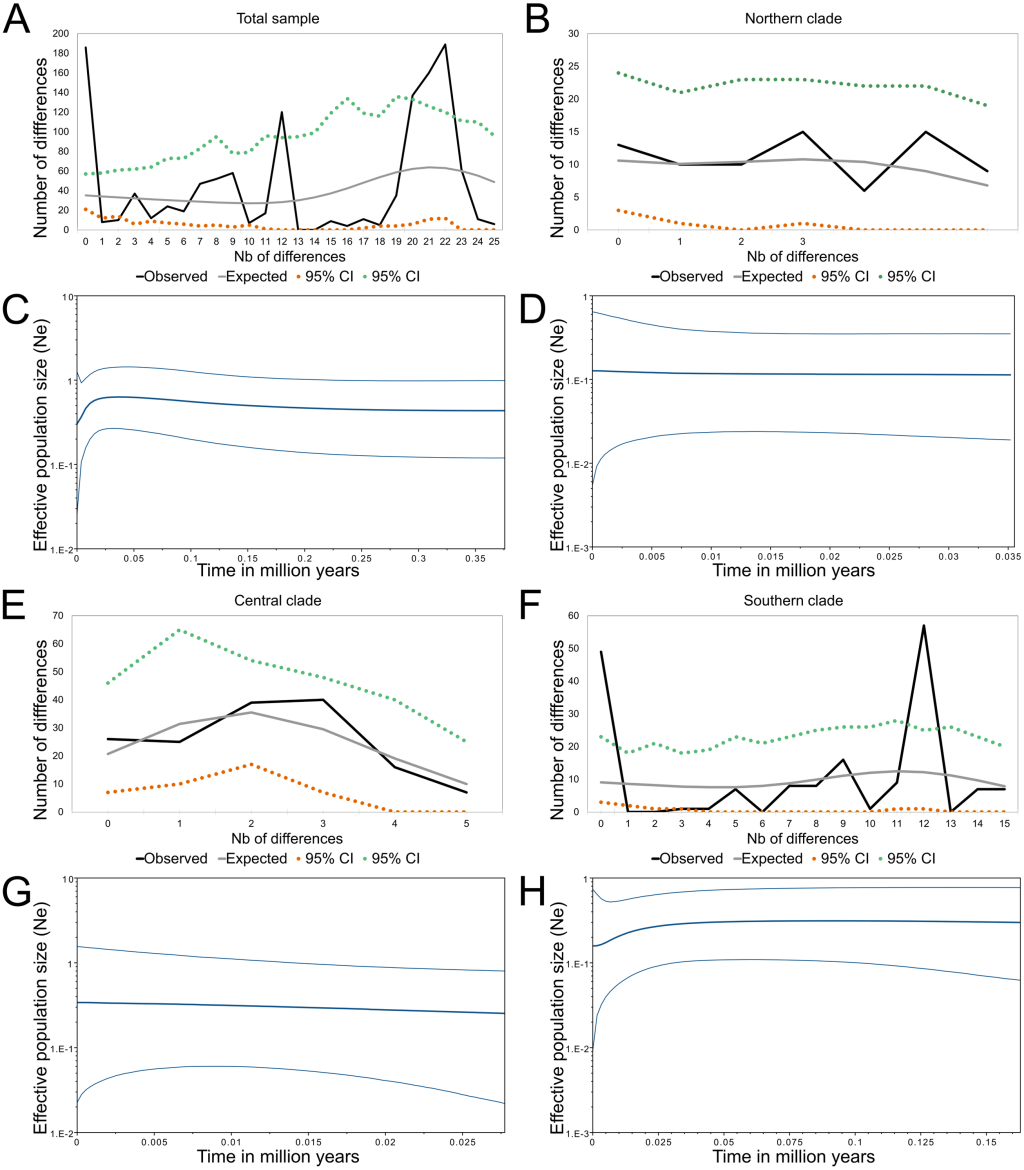
Demographic history of *Abrothrix longipilis.* Mismatch distribution and Bayesian skyline plots for (A), (C) the total sample; (B), (D) Northern clade; (E), (G) Central clade; (F), (H) Southern clade.

The demographic oriented analyses of the whole sample of mitochondrial sequences of *Abrothrix longipilis* were contrasting.. Three out of four demographic indexes (Fs, SSD and Hr) suggested demographic expansion, while the fourth, Tajima’s D failed to detect such a pattern (Table 2). Meanwhile, the mismatch distribution showed signals of population stability; moreover, the skyline plot indicated a population reduction that started approximately 125 kyr B.P. when the whole dataset was analyzed (Fig. 4A).

**Table 2:** Demographic history indexes for four arrangements of samples of *Abrothrix longipilis*. Tajima’s D, Fu’s Fs, sum of squared deviation (SSD), Harpending’s raggedness index (Hr), and their respective *p*-values to the right. Values in bold are those that support demographic expansion.

	Group content	*D*	*p*	*Fs*	*p*	*SSD*	*P*	*Hr*	*p*
Total sample	Localities 1–13	1.19	0.9	**−24.35∼0.00**	**0.05**	**∼0.00**	**0.06**	**0.01**
Northern clade	Localities 1–5	0.51	0.71	**−13.12∼0.00**	0.01	0.65	0.05	0.79
Central clade	Localities 6–8	−1.24	0.11	**−25.87∼0.00**	0.008	0.52	0.03	0.77
Southern clade	Localities 9–13	0.81	0.82	**−13.67∼0.00**	**0.14**	**∼0.00**	**0.29**	**∼0.00**

Both northern and central lineages consistently revealed signals of demographic stability (Table 2). In both cases, only Fu’s Fs indicated demographic expansion. This signal was also consistent with the slight tendency to expansion seen in the Bayesian skyline plots over time (Figs. 4B and 4C). Meanwhile, the southern lineage showed contrasting results. Three out of four demographic indexes supported population expansion (Table 2); the mismatch distribution showed signals of population stability, while the skyline plot indicated a reduction for the most part of the last 30 kyr reaching stability approximately 5–7 kyr B.P. (Fig. 4D).

### Patterns of genetic, geographic, and environmental distances

Patterns of isolation by distance and isolation by environment were detected. Mantel test showed a significant correlation between geographic and genetic distances (*r* = 0.51; *p* = 0.004). With a similar value of r, genetic distance was also significantly correlated with environmental distance (*r* = 0.57, *p* = 0.000).

**Figure 5:**
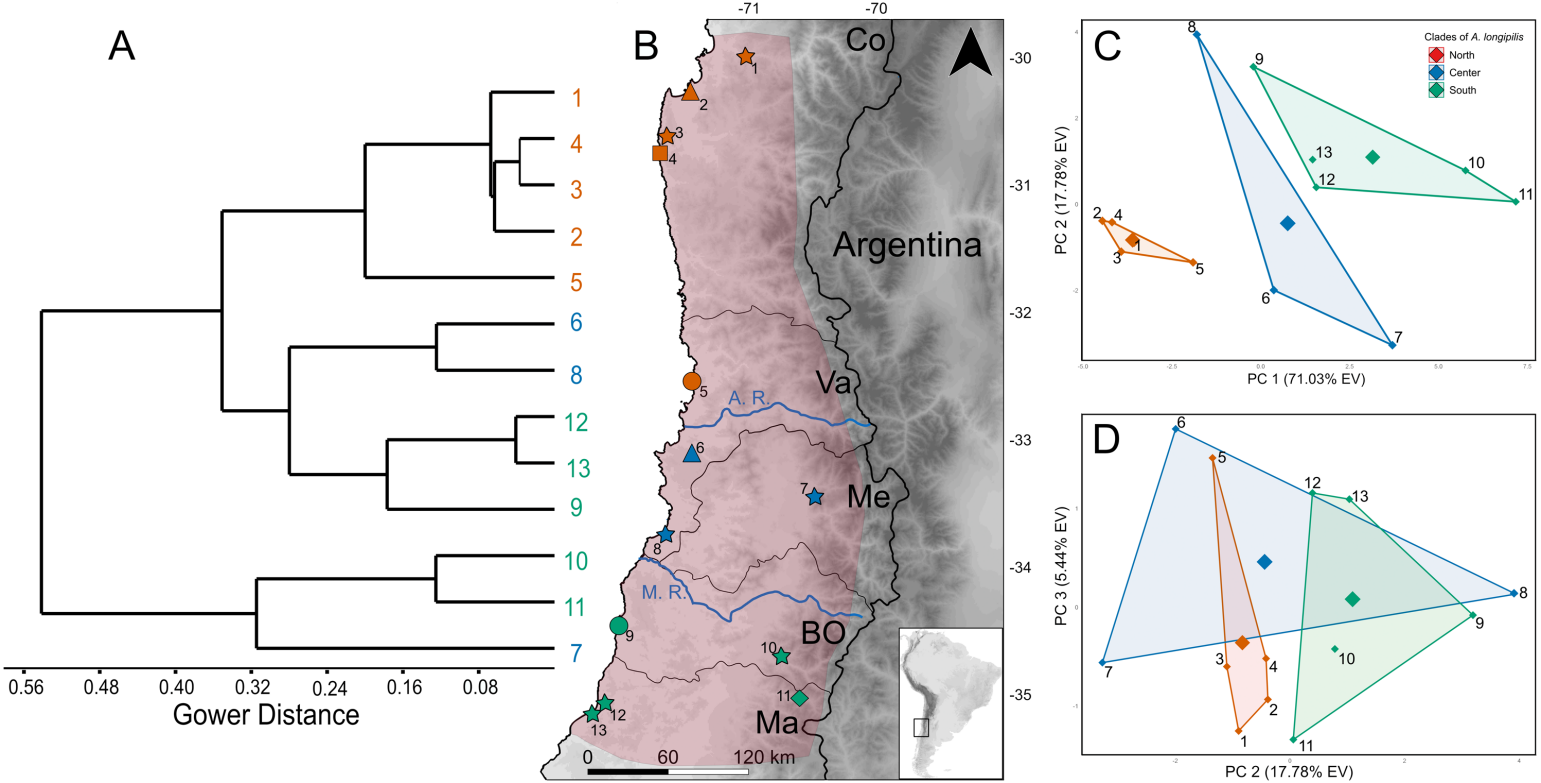
Climatic similarity of the collection localities of individuals of *Abrothrix longipilis*. (A) cluster analysis based on Gower distances of 19 climatic variables of the 13 localities and their location on the map. (B) Symbol and color codes in the map corresponds to that of Fig. 1; locality numbers are those of Table 1. Locations of the rivers Aconcagua (A. R.) and Maipo (M. R.) are shown. (C) PC1 vs PC2 and (D) PC1 vs PC3 derived from the analysis of 19 climatic variables of the collection localities of *A. longipilis*.

The PCA conducted with bioclimatic data showed that the groups of sampling localities where the three mitochondrial lineages of *Abrothrix longipilis* were registered (Fig. 5A), segregate in three distinct areas of the environmental space as delimited by PC1 and PC2 (Fig. 5B). All bioclimatic variables, except Isotermality (Bio 3), had similar loading values for PC1 (Tables S10–S12). PC2 was mainly loaded variables associated with temperature seasonality (e.g., Bio 3, 4, 9 and 10). In the space delimited by PC2 and PC3, localities of the central lineage overlap to those of northern and southern lineages, while the latter two groups did not overlap (Fig. 5C). Finally, in the cluster analysis based on Grower distances, localities formed three main clusters. One of these was formed by the localities corresponding to the northern lineage; the other two clusters mixed localities from the central and southern lineages. Here, instead of segregating in coincidence with mitochondrial lineages, sampling localities formed two groups, one with pre-Andean (7, 10 and 11) and another with coastal (6, 8, 9, 12 and 13) localities (Fig. 5A).

### Ecological niche modelling

**Figure 6:**
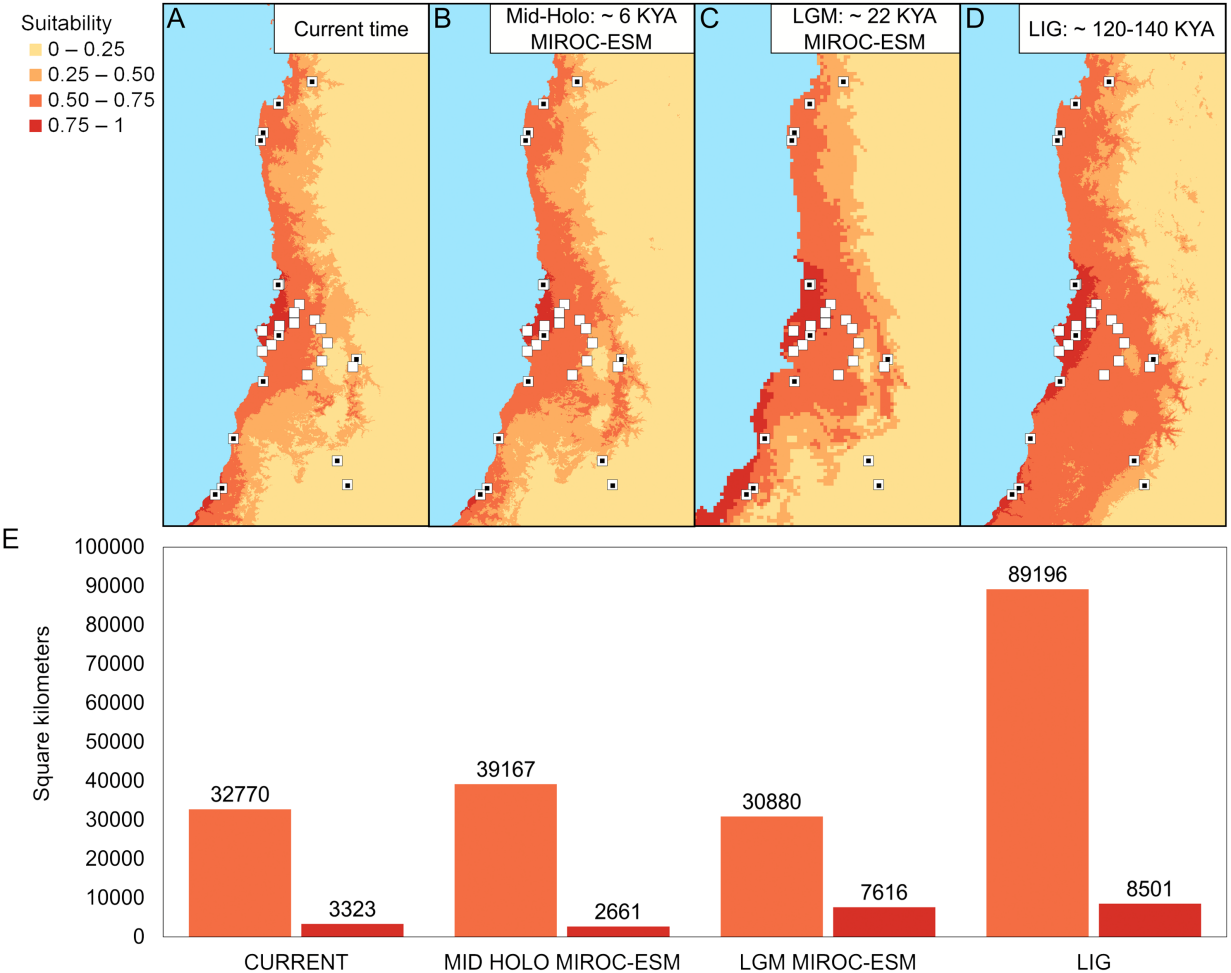
Environmental niche models for *Abrothrix longipilis* at four time points. (A–D) Geographic projections of the models. (E) Areas (in km^2^) of the two upper ranges of habitat suitability (see text for details). White squares indicate localities included in the ENM analyses. White squares with a black circle inside indicate localities from where specimens were sequenced (i.e., those at Fig. 1).

The best configuration of MaxEnt for constructing ENM for *Abrothrix longipilis* was a combination of Linear and Quadratic feature classes and a value of 1 as regularization multiplier (model 4, Table S13). This model yielded values of the area under the curve of 0.89 and omission rate of 0.031, which indicated strong support and good performance. Spatial projections of the ENM on the climatic scenarios evaluated showed that the suitable areas for *A. longipilis* would have experienced reduction from the LIG to the present (Fig. 6). This pattern was more evident in the southern part of the distribution. These projections also showed that the habitat suitability in the central portion of the geographic range remained comparatively more stable across the time span evaluated. Beyond the differences in the projections based on general circulation models (compare MIROC-ESM in Fig. 6A and CCSM4 in Fig. S1), the general pattern depicted was a reduction towards the present in the abiotically suitable areas for *A. longipilis* (Fig. 6B).

## Discussion

The sigmodontine mouse *Abrothrix longipilis* is endemic to the biodiversity hotspot of Central Chile (Myers et al., 2000). The currentlyknown geographic distribution ranges between ca. 30° and 34°S, in the Chilean Regions of Coquimbo, Valparaiso, and Metropolitana (Palma, Cancino & Rodríguez Serrano, 2010; Teta & Pardiñas, 2014). Here we extended the known distribution of this species southwards by 160 and 175 km along coastal (localities 9, 12 and 13) and pre-Andean areas (localities 10 and 11; Fig. 1, Table 1), respectively, to include the O’Higgins and Maule Regions (Fig. 1, Table 1). Additional work is needed to further refine the known distribution of *A. longipilis.* In this regard, maintaining ongoing field collections is essential to reach an adequate knowledge of the Chilean fauna (D’Elía et al., 2019).

In this study, we assessed the genetic variation of *Abrothrix longipilis* using information contained in sequences of the mitochondrial gene *Cytb* and a wide panel of SNPs. RNA-seq remains as a underutilized approach to acquire larger amounts of nuclear sequence data in sigmodontine studies (see Giorello et al., 2018; Prado et al., 2019 for the two so far available studies). In this sense, we are paving the way for the much needed transition to the genomic era of the study of South American rodents (see also Valdez et al., 2015; Giorello et al., 2018), as a way to deepen the understanding of the patterns and processes that underlie rodent diversity (see claims in this line in Lessa et al. (2014) and D’Elía, Fabre & Lessa (2019)).

### Phylogeographic and genetic structure of *Abrothrix longipilis*

The Chilean Mediterranean ecoregion is a biodiversity hotspot; this area harbors a high number of endemics species and high genetic diversity (e.g., Allnutt et al., 1999; Ruzzante et al., 2006; Victoriano et al., 2008; Azpilicueta, Marchelli & Gallo, 2009; Soto, Vidal & Veloso, 2009; Muñoz-Mendoza et al., 2017). *Abrothrix longipilis* is the single sigmodontine species endemic to this region; the co-distributed sigmondontine species (e.g., *Phyllotis darwini*, *A. olivacea*) present wider geographic distributions that reach other ecoregions (see distributional maps in Patton, Pardiñas & D’Elía, 2015). Within this comparatively small distributional area, *A. longipilis* showed a general phylogeographical pattern consisting of three intraspecific lineages that are geographically segregated. We refer to them as the northern, central, and southern lineages (Fig. 1). This pattern is supported by the mitochondrial marker (Fig. 1) and by a wide panel of nuclear SNPs (Fig. 3). The geographic distribution of the three mitochondrial lineages presents a latitudinal rather than altitudinal segregation; in addition, both central and southern lineages are present at coastal as well as pre-Andean areas. It is possible that the northern clade also reaches the Andes but we lack Andean samples at lower latitudes; further field collections are necessary in that area to fill this gap.

The SNP-based PCA provides additional information on the distinction among those lineages, as well as on their internal genetic structure. Consider for instance the location in the PCA of the members of the northern mitochondrial lineage. The mitochondrial genealogy indicated that haplotypes from localities 2, 3, and 5 are more closely related to each other that any of them to samples from other localities. Meanwhile, PC1 vs PC2 and PC1 vs PC3 (Figs. 3A and 3B) also indicated that the genetic similarity of samples from localities 2, 3, and 5 are higher than that to other localities. However, PC2 vs. PC3 showed that localities 2 and 3 are more similar to those from locality 9 (from the southern lineage) than to locality 5. Similarly, samples of the southern lineage, which are similar at PC1 and PC2, fall separately along PC3. In general, SNP dataset provides a finer-grain structure pattern than the mitochondrial gene genealogy; however, given that our geographic sampling for the genomic dataset is poorer than the mitochondrial one, a more dense sampling should be extended to test for consistency. This is of particular interest regarding the southern mitochondrial lineage that distributes in an area of general low suitability (Fig. 6).

Some of the small mammal species that are co-distributed with *Abrothrix longipilis* have been phylogeographically studied. However, as all of them distribute beyond the Mediterranean region, comparisons with *A. longipilis* are not straightforward. We note that the sigmodontine rodents *Phyllotis darwini* and *Oligoryzomys longicaudatus* do not show phylogeographic breaks within our study area (Gutiérrez-Tapia & Palma, 2016; Palma et al., 2014). Other assessed rodents present a latitudinal phylogeographic structure along their complete distributional range, while at the scale of our study (30–35°S) the genetic diversity is segregated longitudinally. This is the case of *Spalacopus cyanus* that exhibits a central coastal clade that differs from an eastern pre-Andean clade (Opazo et al., 2008). Two other small mammals, *Thylamys elegans* and *Octodon degus* also exhibit breaks that are coincident with those we retrieved for *A. longipilis.* The phylogeographic break in *A. longipilis* between the northern+central and southern lineages is concordant with one seen in *T. elegans.* In this species, a northern clade extends from Antofagasta to the Metropolitana Region, while a southern clade distributes from O’Higgins to Maule Regions (Palma et al., 2014). *Octodon degus* also presents two main lineages, whose limits are coincident with those of the northern and central clades of *A. longipilis* (Valladares, 2009). However, the congruence among the breaks of *T. elegans* and *O. degus* with those of *A. longipilis* is only geographical; they differ temporally (3.15 Myr for *T. elegans;* 1.25 Myr for *O. degus*, 230 kyr B. P. for *A. longipilis*). Therefore, the breaks seen in the three species would have arisen due to the effects of different events, which points to a complex regional history.

Phylogeographic studies of other animal and plant species from Central Chile are available in the literature, revealing different genetic patterns and proposing distinct mechanisms to explain them. In some cases involving reptile and amphibian species, a combination of climatic and topographic effects have been invoked to explain population dynamics in Central Chile (e.g., Méndez et al., 2004; Victoriano et al., 2008; Vásquez et al., 2013). For *Aborthrix longipilis*, the Maipo River seems to be a barrier that either originated (primary barrier) or at least maintains (secondary barrier) the divergence between the southern lineage and the northern+central lineage (Fig. 1); additional studies with a narrowed and denser sampling would allow differentiating between both scenarios. The Maipo River was invoked as barrier in other species of plants and animals such as the endemic yam species *Dioscorea humilis* (Viruel, Catalan & Segarra-Moragues, 2014), the frog *Rhinella arunco* (Vásquez et al., 2013), the lizard *Liolaemus manticola* (Torres-Pérez et al., 2007; Muñoz-Mendoza et al., 2017), the snake *Philodryas chamissonis* (Sallaberry-Pincheira et al., 2011), and the mouse opossum *Thylamys elegans* (Palma et al., 2014). None of these papers mentions if the river would be a primary or secondary barrier, but as written, it seems authors favor a scenario invoking a primary barrier. In addition, for the tree *Nothofagus obliqua* (Azpilicueta, Marchelli & Gallo, 2009) and the hemiparasitic plant *Tristerix corymbosus* (Amico & Nickrent, 2009) the Maipo River does not seem to impede gene flow. In addition, the location of the Aconcagua River is coincident with the break that segregates the northern and central lineages of *A. longipilis* (Fig. 1). This river, however, does not seem to affect the gene flow among populations of the frog *Rhinella spinulosus* (Vásquez et al., 2013). It is also possible that the climatic features, in particular the higher aridity to north of the Aconcagua River, contribute to maintain the boundaries between the northern and central lineages of *A. longipilis* more than the river itself. Concordant with this are the results of IBD and IBE patterns (see below).

According to the results of a Mantel test conducted with genetic and geographic distances *Abrothrix longipilis* fit a model of isolation by distance. This result contrasts with those reported for populations of *Spalacopus cyanus* from Central Chile (Opazo et al., 2008), but is coincident with those of *Octodon degus* (Valladares, 2009) in the same area. Alongside with the detection of IBD, *A. longipilis* also exhibits a significant association between its genetic diversity and the climatic features of its geographic range. The three main lineages of *A. longipilis* occupies distinct portions of the Chilean gradient of aridity, which decreases from north to south (Fig. 5). The southernmost site in the northern group (locality 5) is geographically closer to those in the central group (blue points in Fig. 5A) than to the others of sampled sites in the northern group. This is because the northern lineage lies in the Mediterranean desertic-oceanic bioclime, while the rest of the lineages occupy the Mediterranean seasonally rainy-oceanic bioclimatic zone (Luebert & Pliscoff, 2006). Within the latter, the localities where central and southern lineages are found are not markedly distinct (Figs. 5A and 5C). Noticeably, above latitude 33°S, coastal localities (6, 8, 9, 12 and 13) climatically cluster together on one hand, while pre-Andean localities (7, 10 and 12) do the same on the other hand (Fig. 5). However, the genetic diversity of *A. longipilis* is not structured according to climatic features at this spatial scale. Whether intraspecific lineages of *Abrothrix longipilis* presents a niche distinction remains to be tested. To do this, a denser sampling along the whole distributional area, combined with ENM methods will be necessary.

### Historical demography and environmental dynamics

According to our results, the differentiation of the current genetic diversity of *Abrothrix longipilis* started approximately 552 kyr ago, with that of the northern, central, and southern lineages established between 120 and 290 kyr ago. (Fig. 2). Demographic histories of northern and central lineages were characterized by population stability since the LIG period (Table 2, Fig. 4); as such, extant populations of *A. longipilis* currently inhabiting from Coquimbo to the Metropolitana Regions (30°–34°S) have remained relatively stable for the last 25–35 kyr. Population stability in the area was also reported for the subterranean rodent *Spalacopus cyanus* (Opazo et al., 2008). The results of ENM are in agreement with the demographic history of northern and central populations of *A. longipilis*. The areas occupied by northern and central lineages would have remained highly suitable for *A. longipilis* since the LIG to the present (Fig. 6A). Accordingly, the palaeobotanical record shows that in the area there were mostly xerophytic thorn shrubs, sclerophyllous woodland and *Nothofagus* parkland in variable proportions since the mid-Holocene (Villagran, 1995).

Bayesian skyline plots, which lack a probability value for the change at any point, inform about the rate of change in Ne, and the trajectory of this change. Being so, it seems that the southern lineage of *A. longipilis* experienced a demographic reduction ca. 12.5 kyr ago followed by stability for the last 5–10 ky (Fig. 4D). In line with this scenario, ENM showed that the southern areas (33°–35.5°S) were more variable in terms of habitat suitability for *A. longipilis*, particularly for the higher suitability range (0.75–1; see Fig. 6). The suitable southern areas were markedly reduced during the LGM and Mid-Holocene (Fig. 6A). Similar effects of Quaternary climate change during the LGM on the populations of *A. longipilis*, were also inferred for other vertebrates such as *Octodon degus* (Valladares, 2009), *Liolaemus monticola* (Torres-Pérez et al., 2007), and *Rhinella spinulosus* (Vásquez et al., 2013), which also experienced recent demographic expansion in Mediterranean Chile (Fig. 6A).

In the Mediterranean region, central valley and coastal areas were identified as suitable and stable environments for biota during the Quaternary climatic oscillations (Viruel, Catalan & Segarra-Moragues, 2014; Villagran, 1995). Paleopalinological data from the Tagua Tagua lake shows that glacial vegetation persisted at about 34°S up to 10 kyr B.P. (Heusser, 1990). In the case of *Phyllotis darwini* the central valley and coastal hills harboured populations of these species during Pleistocene glacial cycles, colonizing northern, southern, and eastern areas posteriorly from here (Gutiérrez-Tapia & Palma, 2016). However, the Mediterranean area was also colonized after LGM from populations from the north, e.g., *Octodon degus* (Valladares, 2009) or from the south, as it would be the case of *Oligoryzomys longicaudatus* (Palma et al., 2012).

In summary, there are contrasting phylogeographic patterns for the fauna of Central Chile; beyond the particularities of each case, as discussed above, the pattern of *Abrothrix longipilis* is coincident, at least in part, with that of several other species. Most of them exhibit a geographic distribution of their genetic diversity following a latitudinal orientation. In general, the phylogeography of small mammals in Mediterranean Chile depicts two main scenarios: on one hand, a coastal-vs-Andean differentiation, as supported by evidence in *Spalacopus cyanus* and on the other, a latitudinal differentiation, as supported by the patterns of *Thylamys elegans*, *Octodon degus* and the one reported here for *A. longipilis*. These evidence are in line -although they do not exclude other possible explanation- with the model of north to south dispersion of the mammal fauna noted by Moreno et al. (1994), according to which Pleistocene fauna and flora dispersed southwards across opening and closing of corridors along the Andes.

## Conclusions

In this study we extended southwards the known geographic distribution of *Abrothrix longipilis* and found that the species presents three main allopatric mitochondrial lineages that replace each other latitudinally. We showed that historically, populations of *A. longipilis* remained mostly stable through its current distribution. Concordantly, ENM results showed that habitat suitability was relatively stable at most parts of the geographic distribution of *A. longipilis.*

Our study shows the utility of the integration of distinct datasets and analyses to characterize the demographic history of a relatively narrowly distributed mammal species. Similarly, at the time that this study is in the front of the much needed transition of the studies on the evolutionary biology of South American rodents to a genomic era, we expect that the scenario here advanced for *Abrothrix longipilis* be the base when posing the next generation of hypothesis regarding the differentiation of the fauna endemic to the Mediterranean zone of Chile. Finally, by pointing out some of the sampling limitations of our study, we have shown the continuous need to keep doing field collections; this is the common task behind most studies aimed to gain a deeper and complete understanding of the evolutionary aspects of the fauna. This aspect should be understood by the officers in-charge of granting collection permits and supporting research.

##  Supplemental Information

10.7717/peerj.9517/supp-1 Supplemental Information 1 Tables S1-S14

10.7717/peerj.9517/supp-2 Supplemental Information 2 Environmental niche models for *Abrothrix longipilis* at four time points with CCSM4 (A) Geographic projections of the models. (B) Areas (in km2) of the two upper ranges of habitat suitability (see text for details). The model CCSM4 repalces the MIROC-ESM model for the LGM.

10.7717/peerj.9517/supp-3 Supplemental Information 3 Cytb sequences

## Additional Information and Declarations

### Field Study Permissions

The following information was supplied relating to field study approvals (i.e., approving body and any reference numbers): Animal capture and collection were authorized by the Servicio Agrícola y Ganadero de Chile (SAG) under permit numbers 1896/2017, 4532/2017, 1896/2017, 1231/2017, 807/2018 and 1816/2019.

### Data Availability

The following information was supplied regarding data availability: Cytochrome-b sequences are available at GenBank: MN275195–MN275227. These sequences are also available in the Supplementary Files. The raw SNPs data are available at Zenodo: Lourdes Valdez, & Guillermo D’Elía. (2020). Genetic variation of the Chilean endemic long-haired mouse Abrothrix longipilis (Rodentia, Supramyomorpha, Cricetidae) in a geographical and environmental context [Data set]. Zenodo. http://doi.org/10.5281/zenodo.3726537. All specimens that were sequenced were deposited at the Collección de Mamíferos de la Universidad Austral de Chile (the Mammalogy Collection of our University, Universidad Austral de Chile). Specimen numbers: UACH8118, UACH8119, UACH8120, UACH8129, UACH8140, UACH814, UACH8142, UACH8121, UACH8122, UACH8123, UACH8124, UACH8125, UACH8126, UACH8127, UACH8093, UACH8095, UACH8097, UACH8101, UACH8105, UACH8115, UACH8116, UACH8107, UACH8108, UACH8109, UACH8110, UACH8111, UACH8112, UACH8113, UACH8114, UACH8102, UACH8088, UACH8128, UACH8117, and UACH8087.
