# Supplementary figures and images for: Genetic variation of the Chilean endemic long-haired mouse Abrothrix longipilis (Rodentia, Supramyomorpha, Cricetidae) in a geographical and environmental context
Source: PeerJ. 2020 Jul 16;8:e9517. doi: 10.7717/peerj.9517 (PMC7369023; doi:10.7717/peerj.9517)

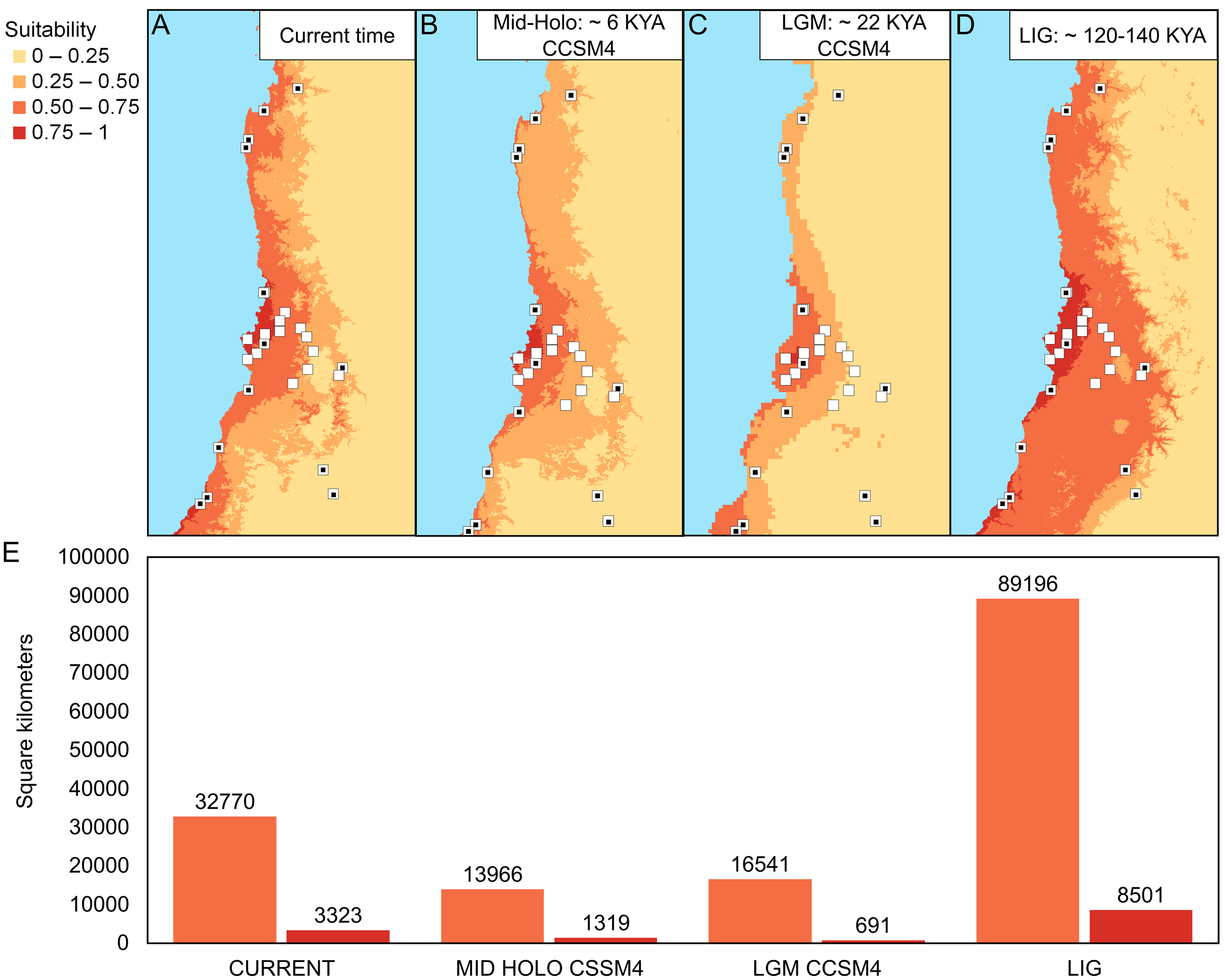

Supplement: Supplemental Information 2 — (A) Geographic projections of the models. (B) Areas (in km2) of the two upper ranges of habitat suitability (see text for details). The model CCSM4 repalces the MIROC-ESM model for the LGM. [file peerj-08-9517-s002.png]
